# (1*E*,4*E*)-1,5-Bis(thio­phen-3-yl)penta-1,4-dien-3-one

**DOI:** 10.1107/S160053681103248X

**Published:** 2011-08-17

**Authors:** S. Shalini, C. R. Girija, Mukesh M. Jotani, B. Rajashekhar, Nageswar Rao, Edward R. T. Tiekink

**Affiliations:** a Chemistry Research Centre, SSMRV College, 4th T Block, Jayanagar, Bangalore 560 041, India; b Department of Physics, Bhavan’s Sheth R. A. College of Science, Ahmedabad, Gujarat 380 001, India; c Department of Chemistry, Sri Sathya Sai Institute of Higher Learning, Andhra Pradesh, Ananthapur 515 134, India; d Department of Chemistry, University of Malaya, 50603 Kuala Lumpur, Malaysia

## Abstract

The title compound, C_13_H_10_OS_2_, exhibits twists between the central C_3_O and ethene residues [O—C—C—C torsion angles = −8.4 (3) and 11.8 (3)°], and between the ethene and adjacent thio­phenyl residues [C—C—C—C torsion angles = −4.2 (3) and 10.5 (3)°]. As a result, the mol­ecule is non-planar, the dihedral angle formed between the terminal thio­phenyl groups being 15.45 (10)°. The presence of C—H⋯O inter­actions involving the bifurcated carbonyl O atom leads to supra­molecular arrays in the *ac* plane. These are linked into a three-dimensional architecture by C—H⋯π inter­actions involving both thio­phenyl residues.

## Related literature

For the use of chalcones in organic synthesis, see: Nehad *et al.* (2007 ▶); Xu *et al.* (2001 ▶). For the biological activity of chalcones, see: Lambert *et al.* (2009 ▶); Boumendjel *et al.* (2008 ▶). Semi-empirical quantum chemical calculations were performed using *MOPAC2009*, see: Stewart (2009 ▶).
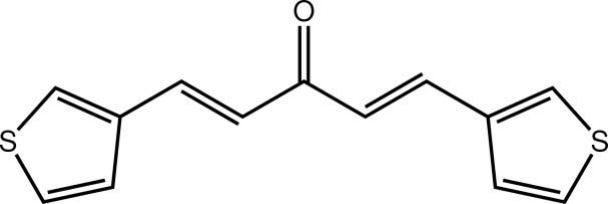


## Experimental

### 

#### Crystal data

C_13_H_10_OS_2_
*M* *_r_* = 246.33
Orthorhombic, 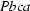

*a* = 11.8908 (3) Å
*b* = 7.1807 (1) Å
*c* = 28.3004 (6) Å
*V* = 2416.41 (9) Å^3^
*Z* = 8
Mo *K*α radiation
μ = 0.42 mm^−1^
*T* = 293 K
0.40 × 0.20 × 0.10 mm

#### Data collection

Bruker SMART APEX CCD diffractometer
39245 measured reflections
2760 independent reflections
2187 reflections with *I* > 2σ(*I*)
*R*_int_ = 0.033

#### Refinement

*R*[*F* ^2^ > 2σ(*F* ^2^)] = 0.040
*wR*(*F* ^2^) = 0.121
*S* = 1.09
2760 reflections
145 parameters
H-atom parameters constrained
Δρ_max_ = 0.33 e Å^−3^
Δρ_min_ = −0.32 e Å^−3^

### 

Data collection: *APEX2* (Bruker, 2004 ▶); cell refinement: *APEX2* and *SAINT* (Bruker, 2004 ▶); data reduction: *SAINT* and *XPREP* (Bruker, 2004 ▶); program(s) used to solve structure: *SHELXS97* (Sheldrick, 2008 ▶); program(s) used to refine structure: *SHELXL97* (Sheldrick, 2008 ▶); molecular graphics: *ORTEP-3* (Farrugia, 1997 ▶) and *DIAMOND* (Brandenburg, 2006 ▶); software used to prepare material for publication: *publCIF* (Westrip, 2010 ▶).

## Supplementary Material

Crystal structure: contains datablock(s) global, I. DOI: 10.1107/S160053681103248X/hg5079sup1.cif

Structure factors: contains datablock(s) I. DOI: 10.1107/S160053681103248X/hg5079Isup2.hkl

Supplementary material file. DOI: 10.1107/S160053681103248X/hg5079Isup3.cml

Additional supplementary materials:  crystallographic information; 3D view; checkCIF report

## Figures and Tables

**Table 1:** Hydrogen-bond geometry (Å, °) *Cg*1 and *Cg*2 are the centroids of the S1,C1–C4 and S2,C10–C13 rings, respectively.

*D*—H⋯*A*	*D*—H	H⋯*A*	*D*⋯*A*	*D*—H⋯*A*
C1—H1⋯O1^i^	0.93	2.49	3.256 (2)	140
C12—H12⋯O1^ii^	0.93	2.44	3.355 (2)	169
C2—H2⋯*Cg*1^iii^	0.93	2.86	3.671 (2)	147
C4—H4⋯*Cg*1^iv^	0.93	2.97	3.809 (2)	151
C11—H11⋯*Cg*2^iv^	0.93	2.83	3.702 (2)	156

Symmetry codes: (i) 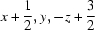; (ii) 
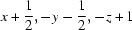
; (iii) 
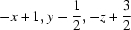
; (iv) 
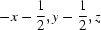
.

**Fig. 1.**
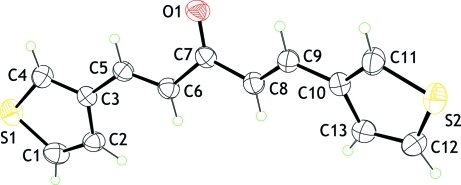
The molecular structure of (I) showing the atom-labelling scheme and displacement ellipsoids at the 35% probability level.

**Fig. 2.**
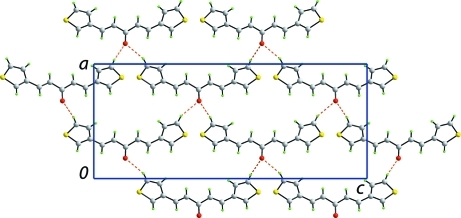
View of the supramolecular array in the ac plane in (I) mediated by C–H···O interactions, shown as orange dashed lines.

**Fig. 3.**
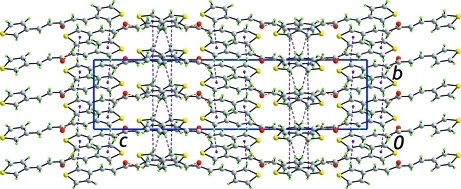
A view in projection down the a axis of the unit-cell contents for (I). The C—H···O and C—H···π interactions are shown as orange and purple dashed lines, respectively.

## supplementary crystallographic information

### Comment

Chalcones attract interest as important chemical intermediates in the synthesis of various organic compounds containing five- (Nehad *et al.*, 2007) and seven-membered (Xu *et al.*, 2001) heterocycles. They also have a wide spectrum of biological activity, *e.g*. as anti-oxidants, neuroprotective, anti-miotics, anti-malarials, *etc*. (Lambert *et al.*, 2009; Boumendjel *et al.*, 2008). In this contribution the synthesis, crystal structure determination and theoretical structure of the title compound, 1,5-bis(3-thiophenyl)-1,4-pentadiene-3-one (I), are reported.

The configuration about each of the ethene [C5═C6 = 1.327 (2) Å and C8═C9 = 1.324 (2) Å] bonds in (I) is *E*, Fig. 1. Small but significant twists in the molecule are observed so that there are notable deviations from planarity. In particular, the carbonyl and ethene groups deviate from co-planarity as seen in the values of the C5—C6—C7—O1 and C9—C8—C7—O1 torsion angles of -8.4 (3) and 11.8 (3) °, respectively. While the S1-thiophenyl ring is effectively co-planar with the adjacent ethene bond [C2—C3—C5—C6 is -4.2 (3) °], the S2-thiophenyl ring is twisted with the C13—C10—C9—C8 torsion angle being 10.5 (3) °. Overall, with reference to the central C_3_O atoms, the thiophenyl groups lie to the same side of the molecule, and form a dihedral angle of 15.45 (10) ° with each other. The conformation of the crystallographic determined molecule structure was subjected to energy minimization calculations using the MOPAC2009 programme with the Parametrization Model 6 (PM6) approximation together with the restricted Hartree Fock closed-shell wavefunction (Stewart, 2009). The minimizations were terminated at a r.m.s. gradient less than 0.01 kJ mol^-1^ Å^-1^. The optimized structure showed that the molecule adopts a non-planar conformation in the gas phase with the dihedral angle between the thiophenyl groups being 9.9 °. A planar arrangement in (I) is precluded owing to the unfavourable H···H interactions that would ensure.

In the crystal packing, the carbonyl-O1 atom plays a prominent role in that it is bifurcated, forming two C—H···O interactions, Table 1. These lead to supramolecular layers in the *ac* plane, Fig. 2. Connections between layers are of the type C—H···π and involve both thiophenyl rings, Table 1. These interactions result in a three-dimensional architecture, Fig. 3.

### Experimental

NaOH (5 g) was dissolved in distilled water (50 ml) and cooled to room temperature. The alkali solution and ethanol (50 ml) were transferred to a 250 ml round bottomed flask. The temperature of the solution was maintained at 298 K and stirred vigorously using a magnetic stirrer. One-half of previously prepared mixture of 0.05 moles of thiophene-3-carboxaldehyde and 0.025 moles of acetone was added to the NaOH-EtOH solution which was then stirred manually. A flocculent precipitate formed within 2–3 minutes of addition. After 15 minutes, the remaining half of the aldehyde-acetone mixture was added to the round bottomed flask, and the mixture was stirred for a further 45 minutes. The solids were filtered under vacuum and washed repeatedly with ice-cold water to eliminate alkali. The solid was pressed between filter paper and dried at room temperature in a desiccator overnight. The compound was recrystallized from EtOH. Yield 82%. *M*. pt. 407–408 K. Colourless needles were obtained by its re-crystallization from hot ethanol solution.

### Refinement

The C-bound H atoms were geometrically placed (C–H = 0.93 Å) and refined as riding with *U_iso_*(H) = 1.2*U_eq_*(parent atom).

### Crystal data

**Table:**

C_13_H_10_OS_2_	*F*(000) = 1024
*M**_r_* = 246.33	*D*_x_ = 1.354 Mg m^−^^3^
Orthorhombic, *P**b**c**a*	Mo *K*α radiation, λ = 0.71073 Å
Hall symbol: -P 2ac 2ab	Cell parameters from 50 reflections
*a* = 11.8908 (3) Å	θ = 5.0–30.0°
*b* = 7.1807 (1) Å	µ = 0.42 mm^−^^1^
*c* = 28.3004 (6) Å	*T* = 293 K
*V* = 2416.41 (9) Å^3^	Needle, colorless
*Z* = 8	0.40 × 0.20 × 0.10 mm

### Data collection

**Table:**

Bruker SMART APEX CCD diffractometer	2187 reflections with *I* > 2σ(*I*)
Radiation source: fine-focus sealed tube	*R*_int_ = 0.033
graphite	θ_max_ = 27.5°, θ_min_ = 1.4°
ω and φ scans	*h* = −15→15
39245 measured reflections	*k* = −8→9
2760 independent reflections	*l* = −36→36

### Refinement

**Table:**

Refinement on *F*^2^	Primary atom site location: structure-invariant direct methods
Least-squares matrix: full	Secondary atom site location: difference Fourier map
*R*[*F*^2^ > 2σ(*F*^2^)] = 0.040	Hydrogen site location: inferred from neighbouring sites
*wR*(*F*^2^) = 0.121	H-atom parameters constrained
*S* = 1.09	*w* = 1/[σ^2^(*F*_o_^2^) + (0.0528*P*)^2^ + 0.9807*P*] where *P* = (*F*_o_^2^ + 2*F*_c_^2^)/3
2760 reflections	(Δ/σ)_max_ = 0.002
145 parameters	Δρ_max_ = 0.33 e Å^−^^3^
0 restraints	Δρ_min_ = −0.32 e Å^−^^3^

### Special details

**Table:**

Geometry. All s.u.'s (except the s.u. in the dihedral angle between two l.s. planes) are estimated using the full covariance matrix. The cell s.u.'s are taken into account individually in the estimation of s.u.'s in distances, angles and torsion angles; correlations between s.u.'s in cell parameters are only used when they are defined by crystal symmetry. An approximate (isotropic) treatment of cell s.u.'s is used for estimating s.u.'s involving l.s. planes.
Refinement. Refinement of *F*^2^ against ALL reflections. The weighted *R*-factor *wR* and goodness of fit *S* are based on *F*^2^, conventional *R*-factors *R* are based on *F*, with *F* set to zero for negative *F*^2^. The threshold expression of *F*^2^ > 2σ(*F*^2^) is used only for calculating *R*-factors(gt) *etc*. and is not relevant to the choice of reflections for refinement. *R*-factors based on *F*^2^ are statistically about twice as large as those based on *F*, and *R*- factors based on ALL data will be even larger.

### Fractional atomic coordinates and isotropic or equivalent isotropic displacement parameters (Å^2^)

**Table:**

	*x*	*y*	*z*	*U*_iso_*/*U*_eq_	
S1	0.38360 (5)	0.12409 (9)	0.833684 (17)	0.0655 (2)	
S2	0.38292 (5)	−0.15855 (9)	0.398067 (18)	0.0658 (2)	
O1	0.17784 (11)	0.0181 (2)	0.61498 (4)	0.0581 (4)	
C3	0.35238 (14)	0.0577 (2)	0.74599 (6)	0.0422 (4)	
C5	0.29957 (15)	0.0511 (2)	0.69974 (6)	0.0441 (4)	
H5	0.2294	0.1072	0.6971	0.053*	
C8	0.33643 (16)	−0.0925 (3)	0.57374 (6)	0.0476 (4)	
H8	0.4061	−0.1500	0.5770	0.057*	
C2	0.45551 (15)	−0.0286 (3)	0.75867 (6)	0.0516 (4)	
H2	0.5002	−0.0944	0.7375	0.062*	
C10	0.34646 (15)	−0.1238 (2)	0.48658 (6)	0.0462 (4)	
C6	0.34072 (15)	−0.0264 (3)	0.66085 (6)	0.0467 (4)	
H6	0.4121	−0.0794	0.6617	0.056*	
C11	0.30593 (18)	−0.0723 (3)	0.44352 (7)	0.0569 (5)	
H11	0.2427	0.0024	0.4395	0.068*	
C9	0.29452 (16)	−0.0692 (3)	0.53080 (6)	0.0472 (4)	
H9	0.2246	−0.0118	0.5288	0.057*	
C4	0.30471 (17)	0.1448 (3)	0.78394 (6)	0.0518 (4)	
H4	0.2366	0.2084	0.7827	0.062*	
C7	0.27673 (15)	−0.0308 (2)	0.61635 (6)	0.0447 (4)	
C1	0.48190 (17)	−0.0050 (3)	0.80489 (7)	0.0616 (5)	
H1	0.5460	−0.0536	0.8191	0.074*	
C13	0.44370 (16)	−0.2385 (3)	0.48152 (7)	0.0550 (5)	
H13	0.4836	−0.2873	0.5069	0.066*	
C12	0.47191 (17)	−0.2690 (3)	0.43574 (8)	0.0616 (5)	
H12	0.5326	−0.3415	0.4261	0.074*	

### Atomic displacement parameters (Å^2^)

**Table:**

	*U*^11^	*U*^22^	*U*^33^	*U*^12^	*U*^13^	*U*^23^
S1	0.0834 (4)	0.0759 (4)	0.0373 (3)	−0.0050 (3)	−0.0023 (2)	0.0014 (2)
S2	0.0802 (4)	0.0740 (4)	0.0433 (3)	0.0006 (3)	0.0076 (2)	−0.0057 (2)
O1	0.0456 (7)	0.0847 (10)	0.0442 (7)	0.0005 (7)	−0.0025 (5)	−0.0016 (7)
C3	0.0453 (9)	0.0400 (8)	0.0411 (8)	−0.0032 (7)	−0.0018 (7)	0.0029 (7)
C5	0.0441 (9)	0.0442 (9)	0.0440 (9)	−0.0013 (7)	−0.0045 (7)	0.0027 (7)
C8	0.0482 (10)	0.0500 (9)	0.0447 (9)	−0.0014 (8)	−0.0005 (8)	−0.0020 (8)
C2	0.0456 (10)	0.0615 (11)	0.0476 (10)	0.0033 (8)	−0.0019 (8)	0.0032 (8)
C10	0.0496 (10)	0.0457 (9)	0.0432 (9)	−0.0017 (8)	−0.0014 (7)	−0.0031 (7)
C6	0.0463 (9)	0.0509 (10)	0.0428 (9)	−0.0003 (8)	−0.0030 (7)	0.0022 (7)
C11	0.0659 (12)	0.0604 (11)	0.0445 (10)	0.0106 (10)	−0.0001 (9)	−0.0047 (8)
C9	0.0485 (9)	0.0485 (9)	0.0447 (9)	0.0013 (8)	0.0004 (7)	−0.0025 (7)
C4	0.0583 (11)	0.0530 (10)	0.0441 (9)	0.0040 (9)	−0.0001 (8)	0.0026 (8)
C7	0.0455 (10)	0.0478 (9)	0.0408 (9)	−0.0068 (8)	−0.0012 (7)	0.0023 (7)
C1	0.0542 (11)	0.0799 (14)	0.0507 (11)	−0.0015 (10)	−0.0095 (9)	0.0139 (10)
C13	0.0482 (10)	0.0634 (12)	0.0535 (11)	0.0015 (9)	−0.0024 (8)	−0.0028 (9)
C12	0.0508 (11)	0.0693 (13)	0.0648 (12)	0.0012 (10)	0.0094 (9)	−0.0111 (10)

### Geometric parameters (Å, °)

**Table:**

S1—C4	1.6982 (19)	C2—H2	0.9300
S1—C1	1.700 (2)	C10—C11	1.362 (3)
S2—C11	1.6960 (19)	C10—C13	1.427 (3)
S2—C12	1.699 (2)	C10—C9	1.450 (2)
O1—C7	1.228 (2)	C6—C7	1.472 (2)
C3—C4	1.366 (2)	C6—H6	0.9300
C3—C2	1.420 (2)	C11—H11	0.9300
C3—C5	1.452 (2)	C9—H9	0.9300
C5—C6	1.327 (2)	C4—H4	0.9300
C5—H5	0.9300	C1—H1	0.9300
C8—C9	1.324 (2)	C13—C12	1.356 (3)
C8—C7	1.468 (2)	C13—H13	0.9300
C8—H8	0.9300	C12—H12	0.9300
C2—C1	1.356 (3)		
			
C4—S1—C1	91.73 (9)	C10—C11—H11	123.6
C11—S2—C12	91.76 (10)	S2—C11—H11	123.6
C4—C3—C2	111.06 (16)	C8—C9—C10	126.70 (17)
C4—C3—C5	122.97 (16)	C8—C9—H9	116.7
C2—C3—C5	125.93 (16)	C10—C9—H9	116.7
C6—C5—C3	127.01 (17)	C3—C4—S1	112.49 (15)
C6—C5—H5	116.5	C3—C4—H4	123.8
C3—C5—H5	116.5	S1—C4—H4	123.8
C9—C8—C7	122.26 (17)	O1—C7—C8	121.58 (16)
C9—C8—H8	118.9	O1—C7—C6	121.05 (16)
C7—C8—H8	118.9	C8—C7—C6	117.36 (16)
C1—C2—C3	112.91 (18)	C2—C1—S1	111.80 (15)
C1—C2—H2	123.5	C2—C1—H1	124.1
C3—C2—H2	123.5	S1—C1—H1	124.1
C11—C10—C13	110.73 (17)	C12—C13—C10	112.92 (18)
C11—C10—C9	123.24 (17)	C12—C13—H13	123.5
C13—C10—C9	126.03 (17)	C10—C13—H13	123.5
C5—C6—C7	121.90 (17)	C13—C12—S2	111.74 (16)
C5—C6—H6	119.1	C13—C12—H12	124.1
C7—C6—H6	119.1	S2—C12—H12	124.1
C10—C11—S2	112.85 (15)		
			
C4—C3—C5—C6	178.38 (18)	C5—C3—C4—S1	178.21 (13)
C2—C3—C5—C6	−4.2 (3)	C1—S1—C4—C3	−0.77 (16)
C4—C3—C2—C1	0.2 (2)	C9—C8—C7—O1	11.8 (3)
C5—C3—C2—C1	−177.47 (18)	C9—C8—C7—C6	−167.02 (17)
C3—C5—C6—C7	177.39 (16)	C5—C6—C7—O1	−8.4 (3)
C13—C10—C11—S2	−0.2 (2)	C5—C6—C7—C8	170.49 (17)
C9—C10—C11—S2	−179.36 (15)	C3—C2—C1—S1	−0.7 (2)
C12—S2—C11—C10	0.47 (17)	C4—S1—C1—C2	0.87 (17)
C7—C8—C9—C10	179.69 (17)	C11—C10—C13—C12	−0.2 (3)
C11—C10—C9—C8	−170.5 (2)	C9—C10—C13—C12	178.87 (18)
C13—C10—C9—C8	10.5 (3)	C10—C13—C12—S2	0.6 (2)
C2—C3—C4—S1	0.5 (2)	C11—S2—C12—C13	−0.61 (18)

### Hydrogen-bond geometry (Å, °)

**Table:**

Cg1 and Cg2 are the centroids of the S1,C1–C4 and S2,C10–C13 rings, respectively.

**Table:**

*D*—H···*A*	*D*—H	H···*A*	*D*···*A*	*D*—H···*A*
C1—H1···O1^i^	0.93	2.49	3.256 (2)	140
C12—H12···O1^ii^	0.93	2.44	3.355 (2)	169
C2—H2···Cg1^iii^	0.93	2.86	3.671 (2)	147
C4—H4···Cg1^iv^	0.93	2.97	3.809 (2)	151
C11—H11···Cg2^iv^	0.93	2.83	3.702 (2)	156

Symmetry codes: (i) *x*+1/2, *y*, −*z*+3/2; (ii) *x*+1/2, −*y*−1/2, −*z*+1; (iii) −*x*+1, *y*−1/2, −*z*+3/2; (iv) −*x*−1/2, *y*−1/2, *z*.
